# The myofascial component of the pain in the painful shoulder of the hemiplegic patient

**DOI:** 10.6061/clinics/2019/e905

**Published:** 2019-04-19

**Authors:** Felipe Martins Liporaci, Márcio Massaro Mourani, Marcelo Riberto

**Affiliations:** Departamento de Biomecanica, Medicina e Reabilitacao do Aparelho Locomotor, Faculdade de Medicina de Ribeirao Preto, Universidade de Sao Paulo, Ribeirao Preto, SP, BR

**Keywords:** Painful Shoulder, Hemiplegia, Treatment, Myofascial Pain Syndrome, Pain, Functioning

## Abstract

**OBJECTIVES:**

Poststroke shoulder pain occurs very frequently and compromises function and quality of life. Because treatment depends on a multidisciplinary approach, it is desirable to optimize effectiveness. Myofascial pain syndrome is defined by the presence of trigger points that can also be found in spastic stroke patients. The aim of this study was to evaluate the frequency of myofascial pain in the shoulder girdle muscles in patients with poststroke shoulder pain and to document the clinical and functional results obtained with specific treatment for this condition.

**METHODS:**

Spastic stroke hemiplegic patients undergoing rehabilitation at the Rehabilitation Center of the Hospital das Clínicas of the Ribeirão Preto Medical School of the University of São Paulo were evaluated regarding the intensity and characteristics of shoulder pain, previous therapeutic interventions, shoulder goniometry and the presence of trigger points. Patients underwent trigger point blockade by intramuscular infiltration of 1% lidocaine. The evaluation and treatment procedures were repeated in the subsequent 3 weeks as long as the pain intensity was greater than 5 on a visual analog scale (VAS). In the fourth week, the evaluation procedures were repeated. Patients who were in a multiprofessional rehabilitation program were instructed to continue the treatment, and the others received complementary therapeutic advice if necessary to initiate it. The evaluations were performed at 0, 1, and 3 weeks and after 4 months.

**RESULTS:**

Twenty-one patients (13 men; age=67.8±10.2 years; right hemiparesis: 11) participated in the study, and there was a reduction in pain assessed by VAS from baseline (7.6±2.7) to the first week (5.8±3.6; *p*<0.05) through the end of the third week (5.2±3.5; *p*<0.05), but not at the end of four months (6.6±2.9; *p*=0.11). Good responders had significantly lower pain levels after the third week and presented with a larger range of motion for passive abduction by the end of 4 months. These results demonstrate that the myofascial component of pain should be considered in poststroke shoulder pain and that its treatment may be a pathway for the rapid and long-lasting relief of symptoms.

**CONCLUSION:**

Trigger point blockade with lidocaine can reduce pain perception in spastic hemiplegic shoulder in as much as 50% of stroke survivors for four months.

## INTRODUCTION

Poststroke shoulder pain (PSSP) is defined by musculoskeletal pain in the weakened shoulder of hemiplegic individuals due to stroke. The clinical features of PSSP vary from mild discomfort to disabling pain, often acting as a precursor factor for secondary anatomical deformities. Pain is most often referred to as heavy, continuous or triggered by passive or active mobilization, is associated with decreased range of motion and increased disability in activities of daily living or care, and can lead to immobility and progressively worsening function. Thus, PSSP interferes with adequate participation in rehabilitation programs 1,2 and is therefore associated with worse prognoses 3-5 and more hospital admissions, where such contexts apply 6. The reported prevalence of PSSP varies in the literature between 16% and 84% 1,5, with significant symptomatology in approximately 70% of cases 1. PSSP usually develops in patients with little or no voluntary movement of the affected upper limb as well as in poorly positioned ones 7. PSSP has been considered an additional risk factor for the onset of depressive disorders, interfering even more in the individual's functioning 3,4.

The etiology of PSSP is still controversial and multifactorial 7, but several factors have been described as causes, such as rotator cuff lesions, glenohumeral dislocation, hand shoulder syndrome, myofascial pain syndrome (MPS), spasticity and contractures, and adhesive capsulitis, among other soft tissue disorders 1,5. Brachial plexus neuropathy secondary to traction also plays a role in the development of unusual patterns of impaired motor function or spasticity or severe focal atrophy. Central pain should also be considered 7,8.

Usually, the condition develops 2-3 months after the stroke, in some cases as early as 2 weeks 2, but usually in the first 6 months 7. Adequate management of PSSP allows the patient to fully participate in the rehabilitation program in addition to promoting better functional outcomes 2. The ideal treatment should start with adequate prevention immediately after the stroke because once the patient presents with pain, secondary anxiety and overprotection can hinder an effective approach 5. The management of PSSP is difficult, and the clinical picture, as well as the responses to the therapies used, is varied, creating a context in which multidisciplinary approaches tend to present a better result. There is little evidence to date regarding the available treatments for this condition.

MPS is a regional pain condition associated with the presence of trigger points (TP). Trigger points are regions of the body surface over the muscles that are painful to palpation and reproduce the patients' complaints, and when properly treated, lead to the improvement of symptoms 9. In these regions, tense muscle bands identified on palpation are associated with a reduced range of motion and sometimes exhibit a brief contraction to the digital motor stimulus or the infiltration needle; this contraction is called a ‘twitch' and denotes the precise identification of the TP. MPS usually occurs in unconditioned muscles, under high tension conditions or after direct local trauma or strains 10. Treatment of this musculoskeletal condition includes the deactivation of TP by procedures such as dry needling or 1% lidocaine infiltration. Additionally, physical resources such as heat and electrotherapy, exercises for the balance of periarticular forces, massage therapy or acupuncture can be used. It is the experience of our group that MPS is a frequent etiology of pain in these cases; thus, the inactivation of TPs in the shoulder muscle girdle can improve PSSP, passive functioning and mobility 11.

### Objectives

This study aimed to evaluate whether TP blockade is effective in reducing pain assessed by VAS and improving shoulder goniometry in spastic subjects with PSSP.

## METHODS

This paper describes a series of twenty-one patients with stable cerebral lesions who were enrolled from an outpatient rehabilitation clinic at the Rehabilitation Center at the Ribeirão Preto General Hospital. After adequate explanations, those who agreed to participate in the study signed the informed consent form recommended by the institutional ethical review board. We based sample size calculations on data from a single study in the literature 11, which documented the effect of TP treatment in this group of patients when compared to the usual treatment, considering a type 1 error of 5% and a type 2 error of 20%.

The inclusion criteria were as follows: either sex; older than 18 years of age; spastic upper limb weakness due to ischemic or hemorrhagic hemispheric stroke; and diagnosis of PSSP, regardless of motor dominance

The exclusion criteria were as follows: previous shoulder pain in the hemiplegic side of the body; pregnant or nursing women; diagnosis of reflex sympathetic dystrophy; presence of pain of central origin; cognitive impairment that impeded assessment and collaboration with treatment; previous adverse reactions to lidocaine; structured joint deformity in the painful shoulder; and uncontrolled coagulation condition.

Procedures: Evaluations occurred at the initial consultation, after one and three weeks and finally after 4 months. The main outcomes were VAS ratings of pain intensity, which ranged from 0 to 10 12, and active and passive goniometry of the shoulder for the movements of abduction and external rotation in the sitting position 13. Additionally, subjects and caregivers answered unstructured questions about the difficulty and pain in performing daily life activities. When the patients could not communicate adequately, the caregivers were asked about it. The muscles that had been systematically examined for this study were the pectoralis major, anterior, lateral and posterior divisions of the deltoid, upper trapezius, supraspinatus, infraspinatus, teres major, subscapularis, biceps brachii, triceps brachii, and brachioradialis, which were identified by superficial palpation according to Travell's guide of TP treatment. Ultrasound or radiological guidance was not required. TP blocks with 1% lidocaine required repetitive movements along the direction of muscle fibers, according to the technique described by Kraus H. 14,15. Needles for this procedure were usually 22G1” (25 x 0.7 mm) in the most superficial muscles; however, 22G1^1/4^” (30 x 0.7 mm) were also used for deeper layers or more obese subjects. Needling was intended to elicit the ‘twitch response', which refers to the localized contraction of part of the muscle fibers that is visible to the naked eye in the superficial muscles. In the deeper muscles, this muscle response is not visible but may be noticed by a movement of the needle for more experienced injectors, and the patient may feel a sharp and subtle sensation that is accompanied by a ‘jump sign’. The mean volume injected was 8.0 ml, and for safety reasons, the maximal volume was not more than 10.0 ml. The therapeutic sessions were repeated for up to 3 consecutive weeks in those patients who indicated pain levels above 5 in the VAS; however, even without this pain level, the subjects were invited to return for all the scheduled evaluations.

Statistical analysis: Qualitative variables were described as percentages in each category, while the quantitative variables were analyzed for normality by the Kolmogorov-Smirnov test and described with adequate measures of central tendency and dispersion. Missing values were imputed using the last observation carried forward technique, and subjects' data were evaluated according to an intention to treat analysis. We used two-way repeated measures ANOVA to test the group*time effect.

Outcomes: Patients who improved at least 4 points on the VAS at any moment after the first TP block were classified as “good responders”, all others as “no response”, and both groups were analyzed separately with the same statistical procedures. The significance level was 5%, with correction for multiple comparisons.

## RESULTS

We recruited 21 patients, of whom 18 fulfilled the inclusion criteria: 13 (72%) were men, the mean age was 66.2±10.5 years and the median time since the stroke was 9.0±6.8 months (time since stroke: good responders: 13.7±11.8 x no response: 7.5±4.0, *p*=0.119). All patients had spasticity in the weakened upper limb, classified as grade 2 or above on the modified Ashworth scale, and 8 had hemiparesis on the right side (Table 1). For 3 patients, only one TP block was performed since there was complete abolition of pain. For 3 other subjects, 2 TP blocks were necessary to achieve sufficient pain relief. Eleven muscles were identified by palpatory surface anatomy in the upper, anterior and posterior portions of the shoulder and arm (Table 1). Myofascial treatment with TP block was most often performed in the subscapularis (30 times), upper trapezius (29 times) and pectoralis major (27 times). Table 2 shows an increase in the passive abduction of the shoulder at the end of the first and third weeks, but not after 4 months; on the other hand, in the active abduction, the gains were minimal and not significant throughout the study. For external rotation, there was no significant change in active or passive goniometry**.**

**Figure 1:**
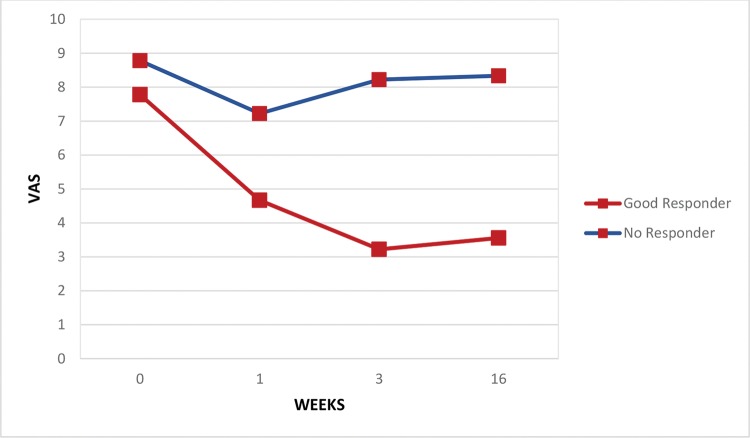
Average pain intensity rating (VAS) of the shoulder over the course of treatment.

**Figure 2:**
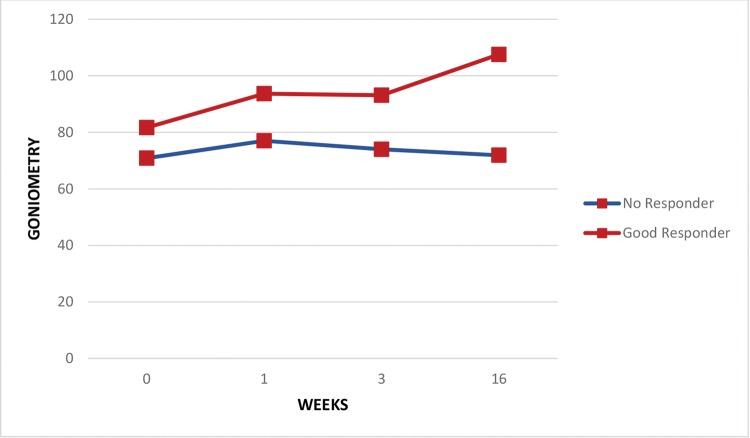
Passive shoulder abduction goniometry based on the patient’s response to treatment.

Regarding the pain assessments, the whole group of patients experienced some relief in the period from the baseline assessment to the end of the first week (VAS scores: 7.6±2.7 and 5.8±3.6 at baseline and after the first week, respectively; *p*<0.05) and through the end of the third week (VAS scores: 7.6±2.7 and 5.2±3.5 at baseline and after the third week, respectively; *p*<0.05) but not at the end of four months (VAS scores: 7.6±2.7 and 6.6±2.9 at baseline and after the fourth month, respectively; *p*=0.11). Both good responders and those with no response had similar pain levels at baseline, but by the third week, pain levels were significantly lower in the good responder group (VAS scores: 8.2±2.2 and 3.2±3.7 at baseline and after the third week, respectively; *p*<0.05) and remained so after 4 months (VAS scores: 8.3±1.6 and 3.5±3.5 at baseline and after 4 months, respectively; *p*<0.05) (Figure 1). Figure 2 shows continuous improvement of passive shoulder abduction in the good responder group, but significant levels were only identified by the end of the follow-up period at 4 months (goniometry: 71.9±22.6 and 107.6±38.2 at baseline and after 4 months, respectively; *p*<0.03). Five subjects were completely asymptomatic with only 2 sessions of TP blocks.

## DISCUSSION

The aim of the study was to evaluate the effects of PSSP treatment by blocking TPs. In agreement with the clinical experience of the researchers, it was possible to confirm the hypothesis that this local inactivation of the TP attenuated the PSSP indicated by improvements in the VAS mean values in the first week after the procedure, with more pronounced results in the third week, and maintenance of pain relief by the end of 4 months. Additionally, when analyzing individual cases, some patients maintained complete abolition or very low pain values throughout the follow-up period. We did not observe any side effects, such as drowsiness, arrhythmia or hypotension, since the administered dose was less than 0.1 g.

It may be argued that other causes of PSSP should have been assessed. Shoulder subluxation is often associated with shoulder pain in stroke survivors, but unless it is a clearly flaccid extremity where the subacromial sulcus is evident, and there is substantial relief with limb support, the literature fails to associate any radiological findings with PSSP. Additionally, adhesive capsulitis is a diagnostic alternative that requires contrast arthrographic findings of a reduction in capsular volume. This condition is clinically suspected when the subject fails to either passively or actively abduct the shoulder, sometimes because of pain. In the spastic hemiplegic patient, failure to abduct the shoulder is usually caused by an increased tone in the adductors, i.e., the pectoralis major, subscapularis, teres major and latissimus dorsi, but it must be stressed that the taut bands in a TP are also associated with reduced range of motion (ROM), thus these three conditions share this same symptomatology. The improvement in ROM in the good responder group in this sample indicated that MPS rather than the other two conditions should be considered, or at least be considered as one component of the pain-related impairment.

The hypotheses of the pathophysiological mechanisms of MPS involve the accumulation of extracellular material that is not reabsorbed after the occurrence of micro- and macro-muscle lesions 16, which results in a limitation of movement and tissue adherence, causing pain, tension, and spasm 17. Shortened and thickened sarcomeres are observed in TPs, while in adjacent regions, the sarcomeres are thin and slender. This generates tension at the TP and in these areas, which can be perceptible in the connective tissues in neighboring structures, causing contractile elements to fail and release substances that sensitize the nociceptors.

Microlesions rupture the sarcoplasmic reticulum and promote the release and accumulation of Ca++ in the sarcoplasm, which interacts with ATP, initiating the interaction of actin and myosin and resulting in the segmental shortening of the sarcomere, which explains the localized hypertonia identified by palpable nodules within the muscle. The exacerbated contraction amplifies the energy consumption that cannot be supplied by the local microcirculation due to the mechanical compression of the capillaries. Consequently, the region starts to work in ischemic conditions, depleting the local ATP and further damaging the active Ca++ uptake. In this way, TPs are associated with prolonged and exaggerated contraction of muscle fibers, causing muscle fatigue. The sum of localized ischemia with extracellular abnormalities and release of algiogenic substances is responsible for the self-sustained vicious cycle of contraction-ischemia-contraction that is responsible for central and peripheral pain sensitization.

Nociceptors linked to nerve fibers III and IV are stimulated and protrude into the posterior horn of the gray matter of the spinal cord, then signals ascend via the rostral projection tracts to supraspinal units. Painful stimuli induce several secondary and CNS-sensitizing alterations contributing to the clinical picture of MPS 18. Spinal cord neurons are spontaneous because of their increased excitability. Therefore, there is allodynia, represented by the increase in the reaction to mechanical stimuli and hyperesthesia caused by the increase in the receptive fields.

Some of the side effects described for TP inactivation with lidocaine infiltration are arrhythmia and hypotension, which are dose-dependent and were not expected to be present in doses as low as those used in this study. Allergic reactions were not observed either. None of the subjects presented adverse events from the needling itself, such as bruises or postprocedure soreness.

Support and proper movement of the shoulder joint depend substantially on the harmony of muscle contraction, which can be severely compromised by the change in strength and tone in stroke individuals. All participants in this study had spastic hemiparesis as the motor sequelae of the brain injury, which may have triggered the onset of TPs due to muscle imbalance and lack of regional harmonic coordination. Considering the reduction in abduction and external rotation in both active and passive mobilization in the initial evaluation, the suspected involved muscles preferentially fell on those with an adductor role and internal rotation, that is, the subscapular, teres major, major dorsal and major pectoral muscles, which share very close insertions in the smaller tubercle and intertubercular groove.

The treatment of MPS can be performed in several ways, all based on the inactivation of TPs: among these treatment modalities are manual inactivation, although there is no evidence of its efficacy 19; nonmanual and noninvasive techniques, among which electrical, mechanical and electromagnetic stimuli are included, for which there is no evidence of their long-term benefits; and finally, the use of invasive techniques, such as dry needling or lidocaine infiltration. The effect of trigger point blockade in PSSP may be due to the mechanical stimulation by the needle or the pharmacological action of topical analgesics (lidocaine). Cummings and White 20 reviewed 21 articles and concluded that the needling efficacy in pain reduction was independent of the drug injected. Hyuk Ga et al. 21 compared needling with acupuncture needles and needling with lidocaine injection and concluded that both treatments were effective and that posttreatment pain was the same in both groups. The systematic review by Lee et al. 1 indicated acupuncture as an effective treatment for PSSP. The most frequently described points to be treated with dry needling are immediately below the acromioclavicular joint or in the shoulder posterior wall, either along the posterior axillary fold posterior to the glenohumeral joint or in the supra- and infraspinal regions, which corresponded topographically to the muscles treated in this study. The treatment effect, in addition to the reduction in pain, was the improvement in sleep quality and a gain in ROM that enabled some daily living activities to become easier, such as dressing, hygiene, or even gait, as well as improvements for caregivers while taking care of their patients. Consequently, adherence to the rehabilitation program was more appealing 2, and there were better functional prognoses 4. Patients with better analgesic responses to treatment are highlighted in Figures 1 and 2 to draw attention to this component of PSSP, which can be easily evaluated in the office and quickly treated with a simple and low-cost procedure with very rewarding results. Statistical comparisons between the subgroups failed to suggest any predictive feature of treatment success. This study was limited by the sample size, which may have reduced the statistical power to demonstrate the results of this therapeutic intervention.

We succeeded in demonstrating the presence of MPS in as many as half of the studied sample, and the proper inactivation of TPs resulted in a reduction in pain and an improvement in ROM. This subgroup of patients with substantial and prolonged therapeutic responses can motivate the clinician to always consider MPS on the list of possible etiologic diagnoses and to master this examination and therapeutic technique, considering the impact that can be quickly obtained to improve functioning, care and quality of life with very little investment.

## AUTHOR CONTRIBUTIONS

Liporaci FM and Mourani MM were the evaluators and the performers of the trigger point blockade and contributed to the manuscript preparation. Riberto M suggested the study, trained the other authors, selected the participants, performed the statistical analysis and supervised the manuscript preparation.

## Figures and Tables

**Table 1:** General characteristics of the subjects.

	Complete sample	Good responders	No response
**n**	18	9	9
**Men (%)**	13	6	7
**Age (years)**	66.2±10.5	67.4±7.9	64.9±13
**Right-handed**	16	7	9
**Right hemiparesis**	9	3	6
**Number of blocks**	2.65	2.4	2.9
**Time since stroke (months)**	9.0±6.8	13.7±11.8	7.5±4.0
**VAS initial**	8.3±1.8	7.8±2.0	8.8±1.5

**Table 2:** Frequency of identification of TP by muscles.

Muscle	Number of blockades	Percentage of blockades
Subscapularis	30	22%
Trapezius	29	21%
Pectoralis major	27	20%
Biceps brachii	11	10%
Supraspinatus	9	7%
Infraspinatus	9	7%
Triceps brachii	7	5%
Deltoid	7	5%
Large dorsal	4	3%
Brachioradialis	2	1.5%
Teres major	1	1%
TOTAL	136	100%
